# Caracterización de β-lactamasas de espectro extendido en aislamientos clínicos colombianos de *Salmonella enterica* no tifoidea de 1997 a 2022

**DOI:** 10.7705/biomedica.6891

**Published:** 2023-09-30

**Authors:** Edna Caterin Rodríguez, Sandra Yamile Saavedra, Lucy Angeline Montaño, Diana Patricia Sossa, Francia Patricia Correa, Jireh Alejandra Vaca, Carolina Duarte

**Affiliations:** 1 Grupo de Microbiología, Instituto Nacional de Salud, Bogotá, D.C., Colombia Instituto Nacional de Salud Bogotá D.C. Colombia

**Keywords:** Salmonella, farmacorresistencia bacteriana, beta-lactamasas, Salmonella, drug resistance, bacterial, beta-lactamases

## Abstract

**Introducción.:**

*Salmonella* spp. es un agente patógeno zoonótico transmitido al humano por el agua o los alimentos contaminados. La presencia de β-lactamasas de espectro extendido es un creciente problema para la salud pública debido a que estas enzimas confieren resistencia contra las cefalosporinas de tercera y cuarta generación.

**Objetivo.:**

Caracterizar las β-lactamasas de espectro extendido en aislamientos de *Salmonella* spp. recibidos por el programa de vigilancia de enfermedad diarreica aguda o enfermedad transmitida por alimentos del Grupo de Microbiología del Instituto Nacional de Salud.

**Materiales y métodos.:**

Entre enero de 1997 y junio de 2022, se recibieron 444 aislamientos de *Salmonella* spp. resistentes, por lo menos, a una de las cefalosporinas de tercera generación. El fenotipo de las β-lactamasas de espectro extendido se identificó con la prueba de doble disco. El ADN se extrajo por ebullición y mediante PCR se amplificaron los genes ^*bla*^ *CTX-M*, ^*bla*^ *SHV y* ^*bla*^ *TEM*.

**Resultados.:**

Todos los aislamientos fueron positivos para la prueba de β-lactamasas de espectro extendido. Los resultados de la amplificación por PCR fueron: ^*bla*^ *CTX-M* + ^*bla*^ *TEM* (n=200), ^*bla*^ *CTX-M* (n=177), ^*bla*^ *SHV*(n=16), ^*bla*^ *SHV* + ^*bla*^ *CTX-M* (n=6), ^*bla*^ *TEM* (n=13) y ^*bla*^ *SHV* + ^*bla*^ *CTX-M* + ^*bla*^ *TEM* (n=3). Del total, 26 aislamientos fueron negativos para los genes evaluados. Los aislamientos positivos para β-lactamasas de espectro extendido se identificaron en Bogotá y en 21 departamentos: Chocó, Magdalena, Meta, Bolívar, Casanare, Cesar, Córdoba, Quindío, Atlántico, Tolima, Cauca, Cundinamarca, Huila, Boyacá, Caldas, Norte de Santander, Risaralda, Antioquia, Nariño, Santander y Valle del Cauca.

**Conclusión.:**

La resistencia a las cefalosporinas de tercera generación en aislamientos de *Salmonella* spp. fue generada principalmente por ^*bla*^ *CTX-M.* El 44 % (197/444) de los aislamientos presentó resistencia a ampicilina, tetraciclina, cloranfenicol y trimetoprim- sulfametoxazol Los serotipos portadores de β-lactamasas de espectro extendido más frecuentes fueron *S*. Typhimurium y *S*. Infantis.

## Abstract (en)

**Introduction.:**

*Salmonella* spp. is a zoonotic pathogen transmitted to humans through contaminated water or food. The presence of extended-spectrum β-lactamases is a growing public health problem because these enzymes are resistant to third and fourth generation cephalosporins.

**Objective.:**

To characterize extended-spectrum β-lactamases in *Salmonella* spp. isolates received by the acute diarrheal disease/foodborne disease surveillance program of the *Grupo de Microbiología* of the *Instituto Nacional de Salud*.

**Materials and methods.:**

A total of 444 *Salmonella* spp. isolates, resistant to at least one of the cephalosporins, were obtained between January 1997 and June 2022. The extended- spectrum β-lactamases phenotype was identified by the double disk test. DNA extraction was carried out by the boiling method, and the ^*bla*^ *CTX-M*, ^*bla*^ *SHV,* and ^*bla*^ *TEM genes* were amplified by PCR.

**Results.:**

All the isolates were positive for the extended-spectrum β-lactamases test. The genes identified were: ^*bla*^ *CTX-M* + ^*bla*^ *TEM* (n=200), ^*bla*^ *CTX-M* (n=177), ^*bla*^ *SHV*(n=16), ^*bla*^ *SHV* + ^*bla*^ *CTX-M* (n=6), ^*bla*^ *TEM* (n=13) and ^*bla*^ *SHV* + ^*bla*^ *CTX-M* + ^*bla*^ *TEM* (n=3). Twenty-six isolates were negative for the evaluated genes. Positive extended-spectrum β-lactamases isolates were identified in Bogotá and 21 departments: Chocó, Magdalena, Meta, Bolívar, Casanare, Cesar, Córdoba, Quindío, Atlántico, Tolima, Cauca, Cundinamarca, Huila, Boyacá, Caldas, Norte de Santander, Risaralda, Antioquia, Nariño, Santander y Valle del Cauca.

**Conclusion.:**

Resistance to third generation cephalosporins in *Salmonella* spp. isolates was mainly caused by ^*bla*^ *CTX-M.* Isolates were resistant to ampicillin, tetracycline, chloramphenicol, and trimethoprim-sulfamethoxazole (44 %; 197/444). The most frequent extended-spectrum β-lactamases-expressing serotypes were *Salmonella* Typhimurium and *Salmonella* Infantis.

Las infecciones por *Salmonella* spp. son una de las principales causas de morbilidad y mortalidad en humanos y animales ^1^. En el mundo, ocurren 93,8 millones de casos de salmonelosis y un estimado de 155.000 muertes cada año ^2^.

Cerca del 34 % de las enfermedades asociadas con agentes patógenos de transmisión alimentaria en Estados Unidos son producidos por *Salmonella* spp. ^3^. *Salmonella* spp. tiene, al menos, 2.600 serotipos adaptados a una amplia variedad de nichos que incluye el intestino de humanos y animales ^4^. La infección en los humanos ocurre por consumo de alimentos contaminados de origen animal, como carne de aves, vacas y cerdos ^5^.

Aunque en la mayoría de los casos la salmonelosis es de resolución espontánea, se puede requerir algún tipo de tratamiento antibiótico para evitar la diseminación de la infección desde el intestino, como el uso de fluoroquinolonas y cefalosporinas ^6^.

La resistencia de *Salmonella* spp. a las cefalosporinas de tercera generación se ha reportado desde 1983 y se ha presentado un incremento de su prevalencia en el mundo ^6^. Las cefalosporinas son una de las principales clases de antimicrobianos de amplio espectro y pueden ser hidrolizadas por β-lactamasas de tipo *Amp* C (como ^*bla*^ CMY-2) y de la clase A en la que se encuentran las β-lactamasas de espectro extendido (^*bla*^ *CTX-M, y* algunos alelos de ^*bla*^ *SHV* y ^*bla*^ *TEM)*^7^.

Al mismo tiempo, se ha incrementado la incidencia de las infecciones causadas por organismos portadores de genes que codifican enzimas de tipo β-lactamasas de espectro extendido, incluyendo *Salmonella* spp., ya que no solo presentan resistencia a los antibióticos de tipo betalactámico, sino también, a otras clases de antimicrobianos. Esto limita las opciones de tratamiento y genera peores pronósticos que las infecciones causadas por cepas no productoras de β-lactamasas de espectro extendido ^8^^,^^9^.

Durante la última década, los genes que codifican β-lactamasas de espectro extendido encontrados con mayor frecuencia, fueron aquellos de la familia de enzimas de tipo *CTX-M,* principalmente portados por plásmidos transferibles y transposones ^10^. La emergencia de β-lactamasas de espectro extendido de tipo *CTX-M* en *Salmonella* spp. ha sido reportada en aislamientos de humanos y animales, y de muestras de alimentos en todo el mundo ^11^^-^^13^. Los plásmidos y transposones que portan los genes *CTX-M* también contienen genes de resistencia a las fluoroquinolonas ^14^.

El incremento de *Salmonella* spp. multirresistente, la elevada prevalencia de enfermedades invasivas en muchas partes del mundo y el inadecuado uso de los antimicrobianos en agricultura para suplir la gran demanda de proteína animal se ha consolidado como un problema de salud pública mundial debido principalmente a la diseminación global de bacterias con mecanismos genéticos de resistencia ^15^^-^^18^.

El objetivo de este estudio fue identificar tres clases de β-lactamasas que producen resistencia a las cefalosporinas de tercera generación en aislamientos de *Salmonella* spp. recuperados de muestras clínicas de pacientes en Colombia.

## Materiales y métodos

Se hizo una búsqueda en la base de datos de *Salmonella* spp. disponible desde enero de 1997 y hasta junio de 2022 (n=15.720), de aislamientos colombianos recuperados por medio del programa de vigilancia por el laboratorio de enfermedad diarreica aguda y enfermedades transmitidas por alimentos. Estos aislamientos fueron remitidos al Grupo de Microbiología (laboratorio nacional de referencia) del Instituto Nacional de Salud, para su caracterización mediante técnicas convencionales de microbiología y paneles automatizados de identificación. La serotipificación se hizo de acuerdo con el esquema propuesto por Kaufmann-White y Le Minor, usando antisueros específicos ^19^.

### Determinación de perfiles de resistencia

Hasta 2014, se utilizaron los paneles del equipo Microscan® (Siemens) para determinar los perfiles de sensibilidad antimicrobiana y obtener datos de concentración inhibitoria mínima para ampicilina (8-16 μg/ml), cefotaxima (2-32 μg/ml), ceftazidima (1-16 μg/ml), ciprofloxacina (1-2 μg/ml) y trimetoprim- sulfametoxazol (1-16 μg/ml - 19-304 μg/ml). Desde el 2015, se empleó la técnica de difusión de disco Kirby-Bauer para tetraciclina (30 pg), cloranfenicol (30 μg), ácido nalidíxico (30 μg), amoxicilina-ácido clavulánico (10 μg), ampicilina (10 μg), cefotaxima (30 μg), ceftazidima (30 μg) y trimetoprim- sulfametoxazol (23,75 μg - 1,25 μg). Para la ciprofloxacina se han utilizado técnicas de microdilución por la prueba de épsilon y por microdilución en placa desde el 2013.

Los resultados fueron validados y analizados con los controles de técnica y los puntos de corte descritos en las recomendaciones del *Clinical and Laboratory Standards Institute y* sus correspondientes actualizaciones anuales ^20^.

### Determinación genética de genes que codifican enzimas tipo β-lactamasas de espectro extendido.

Para la detección de los genes de interés se extrajo el ADN por ebullición y se utilizó la técnica descrita por Monstein *et al.*^21^. Se detectaron fragmentos de 747 pb para ^*bla*^ *SHV*, 445 pb para ^*bla*^ *TEM y* 593 pb para ^*bla*^ *CTX-M.* Las reacciones se llevaron a cabo usando 1 μl de ADN y 10 pmol de cada par de iniciadores en un volumen final de 25 μl. Las condiciones de amplificación fueron: desnaturalización inicial de 95 °C por 15 minutos; 30 ciclos de desnaturalización a 94 °C por 30 s, anillamiento a 60 °C por 30 s, extensión a 72 °C por 2 minutos, y un paso de extensión final de 72 °C por 10 minutos.

## Resultados

### Características de los aislamientos

Se seleccionaron 444 aislamientos de *Salmonella* spp. que presentaban resistencia a cualquiera de las dos cefalosporinas evaluadas: ceftazidima *y* cefotaxima. Se les realizó la prueba de doble disco, según las recomendaciones del *Clinical and Laboratory Standards Institute*^20^ para evaluar la presencia de enzimas tipo β-lactamasa de espectro extendido.

**Figura 1:**
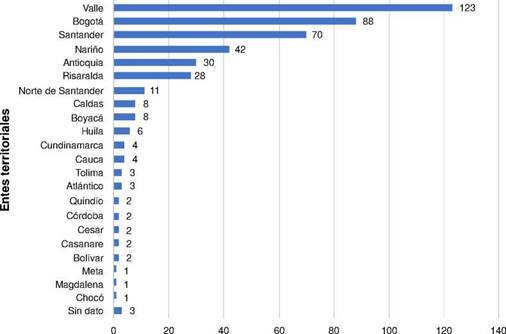
Aislamientos de *Salmonella* spp. positivos para β-lactamasas de espectro extendido, por entes territoriales

Desde 1997 hasta 2013, se remitieron al Grupo de Microbiología menos de 10 aislamientos cada año con resistencia a cefalosporinas de tercera generación. Para el 2014 este número incrementó hasta 24 y fue aumentando cada año incluso hasta llegar a 94 aislamientos postivos para β-lactamasas de espectro extendido en el 2018. Seis departamentos aislaron más de 20 cepas de *Salmonella* spp. con β-lactamasas de espectro extendido, distribuidas así: Valle del Cauca (27,7 %; n=123), Bogotá (19,8 %; n=88), Santander (15,8 %; n=70), Nariño (9,5 %; n=42), Antioquia (6,8 %; n=30) y Risaralda (6,3 %; n=28) (figura 1).

La mitad de los aislamientos con enzimas de tipo β-lactamasas de espectro extendido fueron recuperados de materia fecal (50,9 %; n=226) y los aislamientos restantes se aislaron de hemocultivos (24,8 %; n=110), orina (12,4 %; n=55) y otras muestras invasivas (11,9 %; n=53). Los aislamientos positivos para β-lactamasas de espectro extendido fueron en su mayoría de los serotipos Typhimurium en el 35,4 % (n=157) de los casos, Infantis en el 19,4 % (n=86) y *Salmonella* I 4,[5],12:i:- en el 9 % (n=40). En los 161 aislamientos restantes se encontraron más de 30 serotipos diferentes de *Salmonella* spp.

### *Genes codificadores de* β-lactamasas *de espectro extendido*

Se encontraron siete genotipos diferentes según la presencia de genes que codifican β-lactamasas, el más frecuente fue la combinación *CTX-M* + *TEM* (45 %; n=200), seguido de la presencia única del gen *CTX-M* (40 %; n=177 aislamientos) (cuadro 1).

**Cuadro 1:** Distribución de serotipos y genes codificadores de β-lactamasas de espectro extendido en aislamientos de *Salmonella* spp. de 1997 a 2022

Serotipo	Negativo n (%)	CTX-M n (%)	SHV n (%	TEM n (%	CTX-M + TEM n (%)	SHV+CTX-M n (%)	SHV+ CTX-M + TEM n (%)	Total
Typhimurium	3 (1,9)	17 (10,8)	7 (4,5)	4 (2,5)	118 (75,2)	5 (3,2)	3 (1,9)	157
Infantis	2 (2,3)	73 (84,9)	1 (1,2)		10 (11,6)	-	-	86
I 4,[5],12:i:-	7 (17,5)	3 (7,5)	1 (2,5)	4 (10)	25 (62,5)	-	-	40
Otros serotipos	17 (10,6)	84 (52,2)	7 (4,3)	5 (3,1)	47 (29,2)	1 (0,6)	-	161
Total	29 (6,5)	177 (39,9) 16 (3,6)	13 (2,9)	200 (45)	6 (1,4)		3 (0,7)	444

Los serotipos Typhimurium y I 4,[5],12:i:- presentaron en mayor frecuencia el genotipo *CTX-M* + *TEM*, mientras que en los aislamientos del serotipo Infantis se identificó principalmente el gen de la familia *CTX-M.*

**Figura 2:**
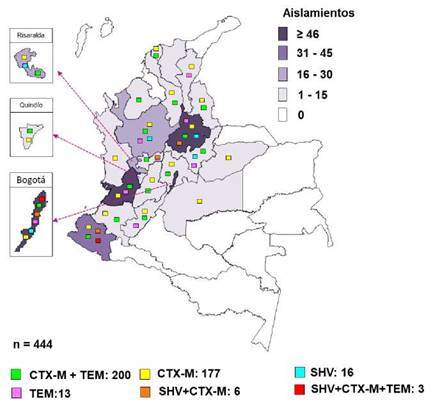
Distribución geográfica según el tipo de mutaciones de β-lactamasas de espectro extendido en *Salmonella* spp. entre enero de 1997 y junio del 2022

Bogotá presentó una proporcion similar de los genotipos *CTX-M* + *TEM y CTX-M* con 39 y 35 aislamientos respectivamente; el genotipo más frecuente de Antioquia y Valle fue *CTX-M* + *TEM,* mientras que en Nariño y Santander fue *CTX-M* (figura 2).

### Perfiles de sensibilidad antimicrobiana

Todos los 444 aislamientos presentaron resistencia a cefotaxima, pero a tetraciclina sólo 88,5 %; a ciprofloxacina, 81,5 %; a cloranfenicol, 73 %; a ceftazidima, 67 %, y a trimetoprim-sulfametoxazol, 59,4 %.

Los dos perfiles de genes codificadores de β-lactamasas de espectro extendido presentaron alta frecuencia de resistencia frente a otros antimicrobianos:

- Genotipo *CTX-M*: los aislamientos con este perfil presentaron resistencia a tetraciclina (88,8 %), ciprofloxacina (89,1 %), cloranfenicol (79,1 %), trimetoprim-sulfametoxazol (57,6 %) y ceftazidima (38,6 %), como se muestra en el cuadro 2.
- Genotipo *CTX-M*+ *TEM:* los aislamientos con este perfil presentaron resistencia a tetraciclina (95,3 %), ciprofloxacina (86 %), cloranfenicol (79,5 %), trimetoprim-sulfametoxazol (71,7 %) y ceftazidima (91,5 %) (cuadro 2).

**Cuadro 2:** Porcentaje de resistencia a los antibióticos según el perfil de genes codificadores de β-lactamasas más frecuente en *Salmonella* spp.

		Genotipo	
Antibiótico	Perfil	***CTX-M* n (%)**	***CTX-M - TEM* n (%)**
Tetraciclina	Resistente	151 (88,8)	182 (95,3)
	Sensible	19 (11,2)	9 (4,7)
Ciprofloxacina	Resistente	131 (89,1)	148 (86,0)
	Sensible	16 (10,9)	24 (14,0)
Cloranfenicol	Resistente	140 (79,1)	159 (79,5)
	Sensible	37 (20,9)	41 (20,5)
Trimetoprim-sulfametoxazol	Resistente	102 (57,6)	75 (71,1)
	Sensible	140 (42,4)	57 (28,9)
Ceftazidima	Resistente	68 (38,6)	183 (91,5)
	Sensible	108 (61,4)	17 (8,5)

## Discusión

La resistencia a antibióticos de *Salmonella* spp. es un problema emergente ya que está limitando las opciones de tratamiento antibacteriano y representa una amenaza considerable para salud pública y la seguridad alimentaria.

En este estudio, el número de aislamientos de *Salmonella* spp. con genes que codifican enzimas de tipo β-lactamasa de espectro extendido se incrementó a lo largo de los años, como lo evidencian las tendencias de resistencia de *Salmonella* spp. reportadas en los informes de vigilancia nacional por laboratorio liderada por el Grupo de Microbiología del Instituto Nacional de Salud ^22^. Una situación similar se reportó en Europa, pues los aislamientos clínicos de *Salmonella* spp. entre el 2018 y el 2019 presentaron altos niveles de resistencia a cefalosporinas de tercera generación, específicamente los serotipos Infantis, Kentucky y Typhimurium ^23^. Esta resistencia se ha relacionado principalmente con la producción de β-lactamasas de espectro extendido y *CTX-M*-1 como el tipo más frecuentemente reportado de β-lactamasa ^23^^,^^24^.

Las enzimas más comunes encontradas en los aislamientos de este estudio fueron de tipo *CTX-M.* Lo anterior se ha consolidado como la tendencia a nivel mundial en aislamientos de *Salmonella* spp. de origen clínico ^25^, así como en otras enterobacterias aisladas de aves de corral ^26^^,^^27^. Estas enzimas son codificadas por genes localizados en plásmidos, lo cual facilita su transferencia horizontal entre diferentes especies y géneros bacterianos ^28^^,^^29^.

Este estudio demostró la presencia de *CTX-M* en varios serovares, incluidos *S.* Typhimurium*, S.* Infantis y *S.* I 4,[5],12:I:-, reportados en otros lugares del mundo ^23^^,^^30^. Esto plantea la preocupación por la transmisión de genes de β-lactamasas de espectro extendido a *S. enterica,* a lo largo de la cadena alimentaria, desde los sistemas de producción primaria a infecciones humanas.

Recientemente, las cepas de *S.* Infantis portadores de β-lactamasas de espectro extendido aisladas de pollos de engorde, carne de abasto y muestras humanas, revelaron un linaje clonal que albergaba un plásmido conjugado especialmente exitoso: *pESl-like plasmid*^8^^,^^31^^-^^33^. La estructura de este plásmido reporta genes de β-lactamasas de espectro extendido (^*bla*^ *CTX-M*-65 y ^*bla*^ *CTX-M*-1), así como otros genes de resistencia -*tet*(A), *sul*1, *dfr*A1, *dfr*A14, *aad*A1- y resistencia a metales pesados ^34^. El proceso específico de adaptación de este plásmido observado en *S*. Infantis, puede facilitar la diseminación de resistencia en aislamientos de *Salmonella* spp. ^35^.

La emergencia y diseminación de este plásmido es preocupante debido a que los aislamientos portadores de este constructo son difíciles de eliminar en infecciones humanas y la presencia de estos genes en elementos genéticos móviles puede facilitar la diseminación de esta resistencia a otros agentes patógenos bacterianos ^33^^,^^34^^,^^36^.

Con respecto a la variante monofásica de *S*. Typhimurium, con fórmula antigénica 1,4,[5], 12:i:- (S. 1,4,[5],12:i:-), se ha observado en años recientes emergencia sanitaria por esta cepa en Europa, siendo la tercera más reportada en aislamientos clínicos del 2019 ^37^. La resistencia a los antimicrobianos de la variante monofásica es un problema emergente de salud pública global. Se ha demostrado que tiene un fenotipo de multirresistencia mayor que otros serovares, con un patrón de resistencia ASSuT (ampicilina, estreptomicina, sulfonamidas y tetraciclina) ^38^.

Durante los años 90, los brotes de enterobacterias productoras de β-lactamasas de espectro extendido ocurridos en todo el mundo, fueron causados principalmente por cepas de *Klebsiella pneumoniae* portadoras de enzimas SHV y TEM. Desde entonces, la resistencia por enzimas CTX-M se ha incrementado rápidamente, ya que las enzimas β-lactamasas de espectro extendido ahora son las más comunes ^39^. En Estados Unidos, las enterobacterias productoras de β-lactamasas de espectro extendido son un problema común en los centros de atención de salud. En 2017, estos agentes patógenos causaron cerca de 200.000 infecciones, desencadenando 9.000 muertes y costos de tratamiento cercanos a los USD $1,2 billones ^5^.

Se ha demostrado que el incremento de las β-lactamasas de espectro extendido puede derivarse del uso de cefalosporinas en la agricultura y en la salud animal. En estudios recientes en China, se han reportado frecuencia elevadas de recuperación de *Salmonella* spp. con β-lactamasas de espectro extendido a partir de alimentos (9,7 %) y de carne de animales (17,7 %) ^13^^,^^40^.

El hallazgo de aislamientos de cepas de *Salmonella* spp. portadoras de β-lactamasas de espectro extendido, se reporta frecuentemente en alimentos listos para el consumo, por lo que son una fuente potencial de diseminación de agentes patógenos a partir de la cadena alimentaria ^41^. En el presente estudio no se estableció una relación entre las β-lactamasas de espectro extendido encontradas en aislamientos clínicos humanos y las presentes en los alimentos o en el campo agrícola. Sin embargo, existe evidencia de que el uso generalizado de cefalosporinas de espectro extendido en medicina humana y veterinaria, combinado con la aplicación no terapéutica en la agricultura, está impulsando la propagación de mecanismos genéticos de resistencia a los antimicrobianos en agentes patógenos transmitidos por los alimentos en todo el mundo ^18^^,^^42^.

Lo anterior resalta la necesidad de fortalecer la vigilancia de la resistencia a los antimicrobianos en *Salmonella* spp., combinada con un manejo adecuado de antibióticos con enfoque *One Health,* con el fin, por ejemplo, de preservar la ceftriaxona para el tratamiento de salmonelosis humana grave. Para esto es necesario entender que la resistencia antimicrobiana está influenciada por el uso de antibióticos en el tratamiento de infecciones humanas y en los ámbitos veterinario, agrícola y ambiental ^43^^,^^44^. Otro ejemplo del abordaje *One Health,* para preservar la efectividad de estos antibióticos es la directriz de la US *Food and Drug Administration* que, desde el 2012, prohibió el uso de cefalosporinas en ganado vacuno, porcino y avícola (pollos y pavos) ^45^.

En un análisis de aislamientos de *Salmonella* spp. de la cadena productiva avícola en Colombia, llevado a cabo entre el 2008 y el 2013, determinó que los genes de tipo β-lactamasas de espectro extendido- AmpC tipo ^*bla*^ *CMY-2*, ^*bla*^ *CTX-M-165*, ^*bla*^ *SHV-12* y ^*bla*^ *SHV-129*, eran los más prevalentes en aislamientos resistentes a cefalosporinas de tercera generación ^46^. Aunque los serovares encontrados en las aves de este estudio colombiano son diferentes a los reportados en casos humanos, su presencia en plásmidos alerta sobre una posible transferencia horizontal a aislamientos más relacionados con infecciones humanas y, con ello, un potencial desenlace inadecuado del tratamiento.

Los resultados obtenidos en el presente estudio respecto al incremento de aislamientos de *Salmonella* spp. portadoras de genes codificadores de β-lactamasas de espectro extendido, ponen de manifiesto la necesidad de integrar la vigilancia de este agente enteropatógeno, para monitorear las tendencias de resistencia a los antibióticos. Lo anterior, con el fin de detectar agentes patógenos resistentes emergentes en infecciones humanas o en animales destinados a consumo humano ^5^^,^^11^^,^^16^^,^^47^.

Asimismo, se evidencia la necesidad de generar políticas e intervenciones locales, nacionales y transnacionales coordinadas, para regular la administración de antimicrobianos en la medicina humana y en la producción de alimentos, tal como se articula en el plan de acción global coordinado por la Organización Mundial de la Salud ^48^.

En Colombia, la vigilancia por laboratorio de este agente patógeno permite conocer la distribución de sus patrones de resistencia a los antimicrobianos y los factores genéticos de *S*. *entérica*. No obstante, presenta limitaciones en lo que respecta a la determinación de la carga de enfermedad por cepas de *Salmonella,* portadoras de β-lactamasas de espectro extendido, que son transmitidas por alimentos. Esto se debe a que la vigilancia es pasiva y a que se lleva a cabo una selección de aislamientos con características de resistencia específicas.
